# Role of dietary interventions on microvascular health in South-Asian Surinamese people with type 2 diabetes in the Netherlands: A randomized controlled trial

**DOI:** 10.1038/s41387-024-00275-5

**Published:** 2024-04-10

**Authors:** Anouk I. M. van der Velden, Daphne H. T. IJpelaar, Prataap K. Chandie Shaw, Hanno Pijl, Hans Vink, Johan van der Vlag, Ton J. Rabelink, Bernard M. van den Berg

**Affiliations:** 1 Department of Internal Medicine (Nephrology), Leiden University Medical Center, Leiden, The Netherlands; 2 Einthoven Laboratory of Vascular and Regenerative Medicine, LUMC, Leiden, The Netherlands; 3 Department of Internal Medicine and Nephrology, Green Heart Hospital, Gouda, The Netherlands; 4 Department of Internal Medicine and Nephrology, Haaglanden Medical Center, The Hague, The Netherlands; 5 Department of Internal Medicine (Endocrinology), LUMC, Leiden, The Netherlands; 6 Department of Physiology, Cardiovascular Research Institute Maastricht, Maastricht, The Netherlands; 7 MicroVascular Health Solutions LLC, Alpine, Utah USA; 8 Department of Nephrology, Radboud University Medical Center, Nijmegen, The Netherlands

**Keywords:** Randomized controlled trials, Cardiovascular diseases

## Abstract

**Background/objectives:**

We investigated whether dietary interventions, i.e. a fasting mimicking diet (FMD, Prolon®) or glycocalyx mimetic supplementation (Endocalyx^TM^) could stabilize microvascular function in Surinamese South-Asian patients with type 2 diabetes (SA-T2DM) in the Netherlands, a patient population more prone to develop vascular complications.

**Subjects/methods:**

A randomized, placebo controlled, 3-arm intervention study was conducted in 56 SA-T2DM patients between 18 and 75 years old, for 3 consecutive months, with one additional follow up measurement 3 months after the last intervention. Sublingual microcirculation was assessed with SDF-imaging coupled to the GlycoCheck^TM^ software, detecting red blood cell velocity, capillary density, static and dynamic perfused boundary region (PBR), and the overall microvascular health score (MVHS). Linear mixed models and interaction analysis were used to investigate the effects the interventions had on microvascular function.

**Results:**

Despite a temporal improvement in BMI and HbA1c after FMD the major treatment effect on microvascular health was worsening for RBC-velocity independent PBR_dynamic_, especially at follow-up. Glycocalyx supplementation, however, reduced urinary MCP-1 presence and improved both PBR_dynamic_ and MVHS_dynamic_, which persisted at follow-up.

**Conclusions:**

We showed that despite temporal beneficial changes in BMI and HbA1c after FMD, this intervention is not able to preserve microvascular endothelial health in Dutch South-Asian patients with T2DM. In contrast, glycocalyx mimetics preserves the microvascular endothelial health and reduces the inflammatory cytokine MCP-1.

**Clinical study registration:**

NCT03889236.

## Introduction

Diabetic vascular complications account for an enormous health burden worldwide with various ethnic groups that are more prone to develop such vascular complications [1, 2]. In the Netherlands, people from South-Asian Surinamese descent are characterized by such high vascular vulnerability, resulting in increased prevalence of micro- and macrovascular complications in diabetes [3–5]. At time of diagnosis these higher rates of complications are already found [3, 6, 7] and progression is also much faster compared to other ethnic groups, translating into a 40 times higher risk for end stage renal disease [8] and a 50% higher age-adjusted mortality rate from coronary heart disease [9].

One of the first hallmarks of vascular damage is endothelial dysfunction, which can progress to structural microvascular changes, and eventually result in irreversible vascular damage [10–14]. Upregulation of glycocalyx degrading enzymes and inflammatory cytokines such as heparanase-1 (HPSE-1) and monocyte chemoatracctant-1 (MCP-1) augments endothelial dysfunction by impairing the endothelial glycocalyx (EG), a mesh of glycosaminoglycans (GAGs), proteoglycans, glycoproteins and glycolipids on the apical side of endothelial cells [15]. Next to conventional therapeutic options, preserving endothelial function in South Asian patients with T2DM (SA-T2DM) could prevent or reduce the fast progression of vascular complications through inhibiting glycocalyx degrading enzymes or reducing metabolic risk factors that contribute to glycocalyx degradation.

Intermitting fasting or fasting mimicking diets (FMD) have been shown to be able to induce cellular changes that affect inflammation and cellular protection [16–21]. A study of T2DM patients with micro-albuminuria showed that FMD could be conducted safely and showed beneficial effects on albuminuria [22]. Recently, we revealed in an experimental diabetic study that repeated FMD partially preserved the glomerular endothelial glycocalyx coverage, however, perturbing glomerular metabolic responses [23]. Also a new promising dietary intervention through supplementation with GAG mimetics such as fucoidan, a marine organism-derived fucosylated and sulfated polysaccharide and major constituent of Endocalyx^TM^, has been shown to preserve the EG layer [24, 25].

Here, we investigated the effects of these two dietary interventions, repeated FMD cycles or Endocalyx^TM^ supplementation in SA-T2DM in a multi-arm randomized controlled study. We hypothesized that dietary intervention is able to preserve endothelial stability and in turn preserve microvascular health.

For this, microvascular health was assessed on the sublingual microvasculature with the non-invasive side-stream darkfield (SDF) imaging method with newly developed and validated Glycocheck^TM^ software [26, 27]. In addition, besides demographics, medication use, blood pressure and laboratory markers for diabetes and renal function testing, we measured the HPSE-1 and hyaluronidase 1 (HYAL-1) activity, and HYAL-4 activity and concentration, together with the endothelial activation markers angiopoietin-2 (ANG2), soluble thrombomodulin (sTM) and MCP-1.

## Research design and methods

### Clinical study design and patient recruitment

**Fig. 1:**
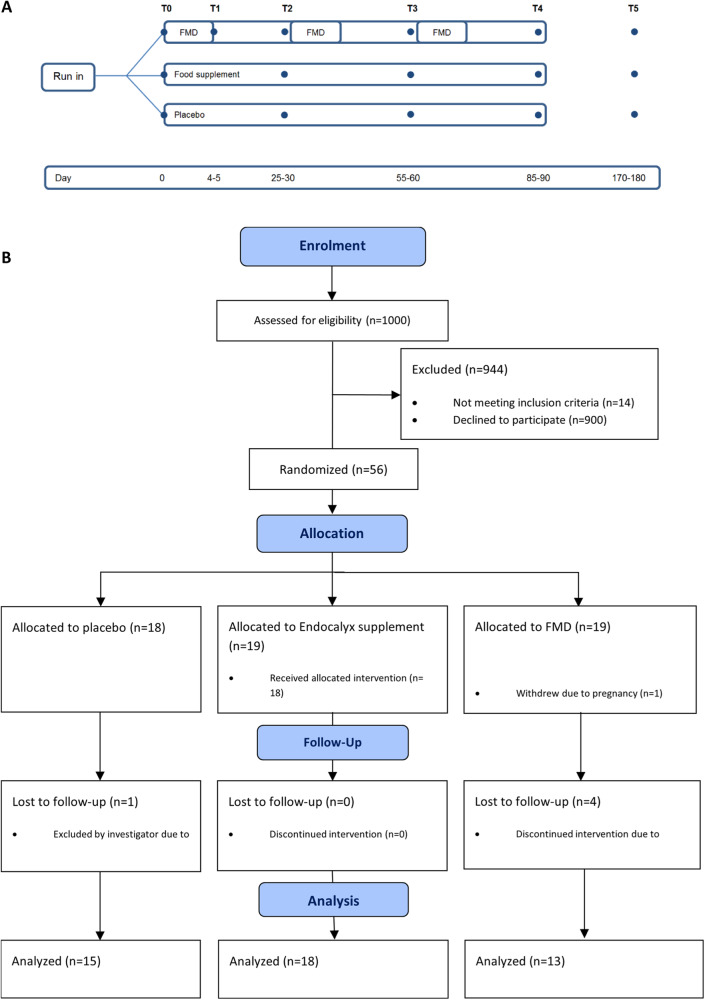
Study design and patient inclusion. Study design (**A**) and CONSORT Flow diagram (**B**).

We conducted a multi-arm parallel-group randomized study in SA-T2DM of Surinamese descent in The Hague area of the Netherlands between May 2018 and September 2020, recruited at general practitioners’ offices. In this study 3 parallel arms were selected: 1) receiving a diet regime of FMD boxes (Prolon®, L-Nutra Inc., Los Angeles, CA, USA); 2) receiving the dietary supplement Endocalyx^TM^ (MicroVascular Health Solutions LLC, Alpine, UT, USA); or 3) receiving placebo capsules (Fig. 1A). For sample size estimation the primary outcome was determined as improvement of the Microvascular Health Index MVHS_dynamic_ according to a pilot study with 13 healthy volunteers receiving the food supplement Endocalyx^TM^ for 3 consecutive months (supplementary Fig. 1). Eligible patients, with inclusion criteria of age between 18 and 75 years old, self-identified as from South-Asian Surinamese descent, treatment with hypoglycemic drugs for type 2 diabetes and proven albuminuria with an albumin to creatinine ratio (ACR) between 0.3 and 30 mg/mmol in the last 12 months. Moreover, eGFR had to be above 45 *mL/min/*1.73 m^2^ (CKD-EPI formula [28]). The study (NCT03889236) was conducted in accordance with the Declaration of Helsinki (October 2013) and approved by the Ethics Committee of the Leiden University Medical Center (LUMC) in agreement with the Dutch law for medical research involving human subjects.

### Intervention, randomization and blinding

Eligible patients were randomized via randomization envelopes made by the Pharmacy department of the LUMC into the diet-, supplement-, or placebo-arm, after given informed consent (Fig. 1). In agreement with the CONSORT statement, randomisation was performed to provide blinding of the supplement and placebo arm to the participants, care providers and researchers. The Pharmacy department redistributed, labeled and blinded the capsules.

FMD (Prolon®) consisted of a 5-day low protein plant-based diet regime that contained energy bars, vegetable-based soups, kale chips, olives, energy drinks, a supplement and natural tea’s. Day 1 provided 1090 kcal (containing 34% carbohydrate, 56% fat and 10% protein), and days 2 to 5 were identical in formulation and provided 725 kcal (47% carbohydrate, 44% fat and 9% protein). As previous studies with this diet showed beneficial effects after 3 monthly cycles [16], our patients also followed the diet once a month, with a total of 3 cycles in 3 months. Trajectory patients’ satisfaction about FMD intervention was reviewed with the Diabetes Treatment Satisfaction Questionnaire (DTSQ) [29]. Endocalyx^TM^ supplement was produced and provided by Microvascular Health Solutions (Alpine, UT, USA). One capsule contained fucoidan extracted from *Laminaria japonica* (106.25 mg), glucosamine sulphate (375.0 mg), hyaluronic acid (17.5 mg), a blend of superoxide dismutase and polyphenols (120.0 mg) and stabilizers/bulking agents. The placebo capsules were manufactured by the Pharmacy department of the LUMC and contained microcrystalline *cellulose*. Patients were instructed to take 4 capsules a day for 3 consecutive months. Patients received no dietary advice and maintained their normal diet during the study.

### Data collection

Study visits were conducted at the general practitioner’s office of that specific patient and were executed by the researcher or research assistants. The web based relational database management system Castor Electronic Data Capture (*EDC*) (https://www.castoredc.com) was used for data storage. Patients were instructed to fast overnight and not to smoke before each study visit. In the first three months, patients had a study visit each month. After completing the first 3 months of the intervention, the interventions were discontinued, and patients had one follow-up study visit at month 6 (Fig. 1A).

At baseline, self-identified ethnicity, age and smoking status were collected. Medical history and medication use was extracted from the personal health records. Microvascular complications were defined as having retinopathy and/or neuropathy. Macrovascular complications were defined as having myocardial infarction, angina pectoris, cerebrovascular accident and/or peripheral artery disease.

Systolic and diastolic BP was measured twice with an automated blood pressure monitor (OMRON, Model M6, Omron Health Care Inc, IL, USA) after patients were sitting calmly for about 5 min. BMI was calculated by dividing the weight (measured with indoor clothing but without shoes) in kilograms by the self-reported height in meters squared. Waist circumference was measured with a measuring tape mid-way between the lower costal margin and the iliac crest. Fasting blood glucose levels were measured with a finger prick blood sample (Accu-chek Aviva, Roche, Basel, Swiss).

Blood samples were collected after overnight fast at the morning of the study visit at baseline, 3- and 6 months through vena puncture. Serum levels of C-peptide, Insulin growth factor 1 (IGF-1), creatinine, high sensitivity C-reactive protein (HsCRP), total cholesterol, high-density lipoprotein cholesterol, triglycerides levels and plasma levels of HbA1c were determined in the central clinical chemistry laboratory of the LUMC using standard assays. Low-density lipoprotein cholesterol was calculated using the Friedewald formula [30]. The CKD-EPI formula was used to estimate the glomerular filtration rate [28].

Plasma HPSE-1 (heparan sulfate, HS degradation) activity (Takara Bio Inc., Shiga, Japan), HYAL-1 [31] (chondroitin sulfate/hyaluronan, CS/HA) and HYAL-4 [32] (CS) activity in-house developed ELISA [33], optimized by use of recombinant active human HYAL-1 or HYAL-4 (7358-GH-020 and 6904-GH-020, Bio-techne, Abingdon, UK). HYAL-4 protein was measured (AMS Biotechnology, Abingdon, UK) according to the manufacturer’s instructions. Plasma levels of ANG2 (DANG20, R&D Systems, Abington, UK) and sTM (850.720.096, Diaclone, Besançon, France) were determined as described [32], and measured according to the protocol supplied by the manufacturer.

On the day of study visit, first morning urine was collected to determine albumin and creatinine (CCL, LUMC), albumin-creatinine ratio (ACR) was calculated and for albumin levels lower than 3.0 mg/mL (displayed as <3.0 by CCL), 2.9 was used for ACR calculation. Urinary HPSE-1 activity and MCP-1 concentration were measured (Takara Bio Inc., Shiga, Japan and R&D Systems Europe, Ltd., Abingdon, UK, resp.) according to manufacturer’s protocols and corrected for creatinine concentration.

### Microvascular imaging

Sublingual microcirculation was assessed with SDF-imaging (CapiScope HVCS, KK Technology, Honiton, UK) coupled to the GlycoCheck^TM^ software (Microvascular Health Solutions Inc., Salt Lake City, UT, USA). Image acquisition was automatically mediated through the Glycocheck™ software as described elsewhere [26, 27, 34]. The GlycoCheck™ software detects and extracts the following microvascular parameters: red blood cell velocity(V_RBC_), perfused capillary density, static and dynamic capillary blood volume (CBV), static and dynamic perfused boundary region (PBR), and the overall microvascular health score (MVHS), validated and described earlier [27, 35].

### Glucose monitoring and diet compliance

To minimize the occurrence of hypoglycemia during the diet cycles, dosages of hypoglycemic medications were temporarily altered during the 5 day diet cycle. Sulfonylurea derivatives and short acting insulin were discontinued, long acting insulin 50% reduced, with fasting glucose monitoring on days 6, 7 and 8. Metformin, DPP4 inhibitors, SGLT-2 inhibitors or GLP-1 agonists were continued during the diet cycle. Compliance was checked on the morning of day 5 of the first FMD cycle by measuring fasting ketone body concentration in blood (CareSens Dual, Zkope Healthcare, Sittard, Netherlands) and with ketone sticks in a fresh morning urine sample (Ketostix, Bayer, Leverkusen, Germany). During the cycles patients were contacted by the investigator to check glucose monitoring and compliance with the diet.

### Clinical data and resource availability

Most of the data generated or analyzed during this study are included in the published article (and its online supplementary files). The remainder clinical data generated during and/or analyzed during the current study are not publicly available due to hospital privacy restrictions but can be made available as anonymized data from the corresponding author upon reasonable request.

### Statistical analysis

The primary endpoint was improvement of microvascular function within 3 months as determined with SDF-imaging (capillary density, CBV, PBR and MVH). Secondary endpoints were improvement in clinical parameters (BP, BMI, waist-to-hip ratio), laboratory markers (ACR, fasting glucose, HbA1c, C-peptide, IGF-1, total cholesterol, LDL cholesterol, HDL cholesterol, triglycerides, hsCRP, HPSE-1, HYAL-1, HYAL-4, ANG2, sTM and MCP-1). Potential legacy effects were determined using the microvascular and clinical parameters, 3 months after discontinuation (at month 6).

Continuous variables with normal distribution were presented as mean with standard deviation (SD) and variables with skewed distribution as median with 25–75 percentile. Categorical data were expressed as proportions.

Treatment effects within and between groups (diet vs. placebo and supplement vs. placebo, respectively) were investigated with intention to treat analysis by linear mixed models for repeated measurements with Bonferroni post hoc test (values expressed as estimated marginal means (SE) or estimated mean differences with 95% CI). The models were adjusted for age, gender, microvascular and macrovascular history at baseline and hypertension at baseline as this can influence microvascular function. For the per protocol analysis, delta changes within 3 months intervention were compared between the intervention groups with an unpaired *t* test.

Results from the DTSQ questionnaire (see supplementary data) were compared between FMD and placebo groups with an unpaired *t* test.

Statistical analysis was performed using SPSS version 25 (SPSS Inc., Chicago, IL, USA) and GraphPad Prism version 8 (Graphpad Inc., La Jolla, CA, USA). A significance level of 0.05 was considered statistically significant.

## Results

### Inclusion and drop-outs

A total of 56 patients were included and randomized (see CONSORT diagram in Fig. 1), 19 in FMD group, 19 in Endocalyx group and 18 patients in placebo group. In FMD group, one patient withdrew due to pregnancy at the start of the study and one patient withdrew during the baseline visit due to anxiety for the venipunctures. 3 Patients discontinued due to adverse events or non-adherence during or after the first diet cycle and one patient was withdrawn by the investigator due to an SAE after the first diet cycle. A total of 13 patients completed the 3 diet cycles and the follow-up study visit at month 6 (Fig. 1). In Endocalyx group, 19 patients were randomized and completed the baseline measurement. One patient did not start due to the COVID-19 pandemic, which resulted in 18 patients completing the 3-month intervention and follow-up. In the placebo group, 2 patients withdrew informed consent before the baseline visit and one patient was withdrawn by the investigator after the baseline measurements due to an eGFR below the inclusion criteria threshold. A total of 15 patients completed the 3 months placebo intervention and 14 patients completed the follow-up study visit at month 6, as one patient migrated to Surinam. Due to the COVID-19 pandemic all inclusions had to stop, preventing completion of the estimated number of patients per group.

### Baseline characteristics of the study population

Baseline characteristics of FMD- (*n* = 18), Endocalyx- (*n* = 19) and placebo group (*n* = 16) and total cohort (*n* = 53) are shown in Table 1. Overall, baseline characteristics were almost similar between both dietary intervention groups and placebo group, except mean age in Endocalyx group was younger and mean duration of diabetes mellitus was higher in FMD group compared to placebo group. Several patients in all groups had prevalent micro- or macrovascular diabetes complications. All patients used various combinations anti-diabetes medication. More than half of the patients used antihypertensive medication, of which a large number used renin-angiotensin-aldosterone system (RAAS) inhibitors. Despite this, percentage of patients with hypertension (according to the AHA guidelines ≥130 mmHg systolic or ≥80 mmHg diastolic) was around 89% (FMD), 58% (Endocalyx) and 72% (placebo).

**Table 1:** Baseline characteristics of the study population.

	Placebo (*n* = 16)	Diet (*n* = 18)	Supplement (*n* = 19)	Total (*n* = 53)
**Demographics**				
Age, years (SD)	63 (±7)	61 (±6)	56 (±7)	60 (±7)
Women, *n* (%)	7 (44)	12 (67)	12 (63)	31 (59)
Current tobacco smoking, *n* (%)	5 (31)	4 (22)	6 (32)	15 (28)
**Medical history**				
Duration diabetes mellitus, years (SD)	7 (±5)	11 (±4)	9 (±5)	9 (±5)
Retinopathy, *n* (%)	4 (25)	10 (56)	5 (26)	19 (36)
Neuropathy, *n* (%)	1 (6)	2 (11)	4 (21)	7 (13)
Coronary artery disease, *n* (%)	4 (25)	3 (16)	2 (11)	9 (17)
Angina pectoris, *n* (%)	4 (25)	2 (11)	1 (5)	7 (13)
CVA/TIA, *n* (%)	3 (19)	0	4 (21)	7 (13)
**Medication use**				
Metformin, *n* (%)	16 (100)	17 (94)	19 (100)	52 (98)
DPP4 inhibitor/GLP-1-RA/SGLT2 antagonist, *n* (%)	2 (13)	3 (17)	3 (16)	8 (15)
Sulfonylurea derivatives, *n* (%)	3 (19)	9 (50)	9 (47)	21 (40)
Insulin, *n* (%)	2 (13)	3 (17)	3 (16)	8 (15)
Anti-hypertensive medication, *n* (%)	11 (61)	12 (67)	12 (63)	35 (66)
RAAS inhibitors (*n*)	10 of 11	10 of 12	10 of 12	30 of 35
Statins, *n* (%)	12 (75)	16 (89)	16 (84)	44 (83)
**Blood pressure**				
Systolic blood pressure, mmHg (SD)	139 (±16)	148 (±19)	132 (±15)	140 (±18)
Diastolic blood pressure, mmHg (SD)	86 (±9)	87 (±10)	82 (±11)	85 (±10)
Hypertension^a^, *n* (%)	13 (72)	16 (89)	11 (58)	40 (76)
BMI, kg/m^2^ (SD)	27.4 (±4.3)	28.8 (±5.5)	30.1 (±4.7)	28.8 (±4.9)
**Laboratory markers**				
Fasting glucose, mmol/L (range)	7.3 (±1.6)	7.6 (±1.7)	8.3 (±2.2)	7.8 (±1.9)
HbA1c, % (range) mmol/mol (range)	6.8 (6.5–8.0) 51 (47–63)	7.2 (7.0–7.7) 56 (50–60)	7.2 (6.8–7.9) 56 (51–62)	7.2 (6.7–7.8) 55 (50–61)
Total cholesterol, mmol/L (SD)	3.9 (±0.7)	4.4 (±0.8)	4.4 (±1.1)	4.3 (±0.9)
hsCRP, mg/L (range)	1.6 (0.8–5.2)	2.1 (0.9–5.0)	4.7 (2.9–8.1)	3.2 (1.0–4.7)
eGFR CKD-EPI (ml/min/1.73m^2^)	80 (±17)	84 (±19)	85 (±19)	83 (±18)
**Urinary markers**				
Albumin/creatine ratio (mg/mmol)	1.8 (0.9–3.2)	1.0 (0.6–1.6)	1.0 (0.6–3.9)	1.1 (0.6–2.4)
Normo-albuminuria^b^, (*n*, %)	9 (56)	17 (94)	15 (79)	41 (77)
Micro-albuminuria at baseline^c^ (*n*, %)	7 (44)	0 (0)	3 (16)	10 (19)
Macro-albuminuria at baseline^d^ (*n*, %)	0 (0)	1 (6)	1 (5)	2 (4)

Data is presented as mean (SD), median (25–75 percentile) or number with percentage.

*CVA/TIA* cerebrovascular event/transient ischemic attack, *DPP4* dipeptidyl peptidase-4, *GLP-1-RA* glucagon-like peptide-1 receptor agonist, *SGLT2* sodium-glucose cotransporter 2, *RAAS* renin-angiotensin-aldosterone system, *BMI* body mass index, *HbA1c* hemoglobin A1c, *hsCRP* high sensitivity c-reactive protein, *eGFR CKD-EPI* estimated glomerular filtration rate according to chronic kidney disease epidemiology collaboration

^a^According to the AHA guidelines ≥130 mmHg systolic or ≥80 mmHg diastolic.

^b^Albumin-creatinine ratio between <3.0 mg/mmol. ^c^Albumin-creatinine ratio between 3.0–30 mg/mmol. ^d^Albumin-creatinine ratio > 30 mg/mmol.

Mean BMI was high in all groups, 28.8 ± 5.5 kg/m^2^ (FMD), 30.1 ± 4.7 kg/m^2^ (Endocalyx) and 27.4 ± 4.3 kg/m^2^ (placebo). In general, patients were well regulated as reflected by the average low HbA1c levels. The placebo group had the highest number of patients with albuminuria in the past 12 months (*n* = 7) compared to FMD (*n* = 1) and Endocalyx (*n* = 4).

### Microvascular health in placebo group

First, when combining all baseline data (FMD, Endocalyx and placebo) of measured capillary densities, the percentage capillary density difference in patient group was lower when compared to our earlier published Framingham risk groups in the Netherlands Epidemiology of Obesity (NEO) study (supplementary Fig. 2) [26]. These results reflect the already poor perfused capillary network in South Asian patients with T2DM, compared to the general Dutch population. As the patients in the placebo group did not receive any dietary restrictions it was used as a control for both the dietary interventions. Using linear mixed model analysis adjusted for age and sex revealed that besides the perturbed anatomic vascular structures, endothelial health (PBR, as the inverse glycocalyx dimension) and overall microvascular health score (MVHS_dynamic_) worsened over time in the placebo group (Table 2, supplementary Fig. 3A and supplementary Table 1) [27].

**Table 2:** Differences between baseline and after 3 months in the interventions groups and treatment effects.

	Placebo			Diet			Supplement				
	Baseline	3 months	Difference [95% CI]^a^	Baseline	3 months	Difference [95% CI]^a^	Baseline	3 months	Difference [95% CI]^a^	Treatment effect diet^b^	Treatment effect supplement^b^
	(*n* = 16)	(*n* = 15)		(*n* = 18)	(n = 13)		(*n* = 19)	(*n* = 17)			
**Microvascular parameters**											
Capillary density (4–6) (µm/mm²)	43 (6)	34 (4)	−9 [−23;4]	32 (6)	24 (4)	−7 [−20;6]	32 (5)	35 (3)	3 [−10;15]	0.79	0.11
Capillary blood volume static (pL/mm^2^)/10^3^ μm^3^	12.7 (1.7)	9.9 (1.1)	−2.8 [−6.8;1.3]	10.1 (1.7)	8.1 (1.2)	−2.1 [−6.1;2.0]	10 (1.5)	10.9 (1)	1.0 [−2.8;4.7]	0.76	0.10
Capillary blood volume dynamic (pL/mm^2^)/10^3^ μm^3^	21.6 (2.5)	11.3 (1.4)	**−10.3** [**−16.2;−4.3]**	11.8 (2.5)	14 (1.6)	2.2 [−3.7;8.1]	14.7 (2.3)	13.9 (1.3)	−0.8 [−6.2;4.7]	**0.001**	**0.01**
PBR static (µm)	2.08 (0.05)	2.24 (0.05)	**0.16** [**0.03;0.28]**	2.26 (0.05)	2.2 (0.06)	−0.05 [−0.19;0.08]	2.21 (0.04)	2.18 (0.05)	−0.04 [−0.16;0.08]	**0.01**	**0.01**
PBR dynamic (µm)	2.19 (0.05)	2.46 (0.05)	**0.28** [**0.15;0.40]**	2.35 (0.05)	2.66 (0.06)	**0.32** [**0.18;0.50]**	2.74 (0.04)	2.01 (0.05)	**−0.73** [**−0.85;−0.61]**	0.59	**<0.001**
MVHS dynamic (µm)	3.9 (0.5)	1.8 (0.3)	**−2.1** [**−3.2;−1.1]**	2.1 (0.5)	2.1 (0.3)	0 [−1.0;1.1]	2.2 (0.4)	2.8 (0.2)	0.7 [−0.3;1.7]	**<0.001**	**<0.001**
**Clinical parameters**											
Systolic blood pressure (mmHg)	132 (4)	136 (4)	4 [−5;13]	141 (4)	134 (4)	−7 [−16;3]	133 (3)	130 (3)	−3 [−11;5]	0.05	0.17
Diastolic blood pressure (mmHg)	82 (2)	82 (2)	1 [−5;6]	81 (2)	80 (2)	−2 [−7;4]	80 (2)	81 (2)	1 [−4;5]	0.43	0.95
BMI (kg/m^2^)	26.4 (1.3)	26.4 (1.2)	0 [−0.5;0.4]	28.3 (1.3)	27.3 (1.3)	**−1.0** [**−1.5;−0.5]**	30.2 (1.1)	30.3 (1.1)	0.1 [−0.3;0.6]	**<0.001**	0.53
**Laboratory markers**											
Fasting glucose (mmol/L)	7.3 (0.5)	7.1 (0.6)	−0.1 [−1.4;1.1]	7.2 (0.5)	7.1 (0.6)	−0.2 [−1.5;1.1]	8.3 (0.4)	8.1 (0.5)	−0.2 [−1.3;1]	0.97	0.98
Hba1c (mmol/mol)	55.6 (3.3)	55.9 (3)	0.4 [−3.9;4.6]	55.3 (3.4)	50.3 (3.3)	**−5.1** [**−9.6;−0.6]**	57.9 (2.9)	60.2 (2.7)	2.3 [−1.7;6.3]	**0.03**	0.41
C-peptide (nmol/L)	1.2 (0.1)	1.2 (0.1)	0 [−0.2;0.2]	1.4 (0.1)	1.3 (0.1)	−0.1 [−0.3;0.1]	1.5 (0.1)	1.5 (0.1)	0.1 [−0.1;0.2]	0.47	0.62
eGFR CKD-EPI (ml/min/1.73m^2^)	86 (5)	84 (5)	−2 [−8;5]	83 (5)	81 (5)	−2 [−9;5]	80 (4)	83 (4)	3 [−2;9]	0.97	0.16
Total cholesterol (mmol/L)	4.03 (0.23)	4.12 (0.26)	0.09 [−0.33;0.51]	4.34 (0.24)	4.21 (0.28)	−0.13 [−0.58;0.32]	4.35 (0.2)	4.34 (0.23)	0 [−0.39;0.38]	0.38	0.68
HDL-cholesterol (mmol/L)	1.27 (0.08)	1.35 (0.09)	0.08 [−0.01;0.18]	1.22 (0.08)	1.23 (0.09)	0.01 [−0.09;0.11]	1.08 (0.07)	1.07 (0.08)	−0.01 [−0.09;0.08]	0.20	0.10
LDL-cholesterol (mmol/L)	2.34 (0.2)	2.32 (0.2)	−0.02 [−0.33;0.29]	2.4 (0.21)	2.31 (0.21)	−0.09 [−0.42;0.24]	2.41 (0.18)	2.43 (0.18)	0.02 [−0.27;0.31]	0.70	0.80
Triglycerides (mmol/L)	1.11 (0.5)	1.19 (0.33)	0.07 [−0.6;0.75]	1.65 (0.5)	1.77 (0.35)	0.12 [−0.56;0.8]	2.31 (0.45)	2.09 (0.29)	−0.22 [−0.84;0.4]	0.90	0.43
hsCRP (mg/L)	4.0 (1.1)	3.0 (1.0)	−1.1 [−2.8;0.6]	3.6 (1.1)	2.9 (1.1)	−0.7 [−2.4;1.1]	6.0 (1.0)	6.2 (0.9)	0.2 [−1.3;1.8]	0.68	0.16
IGF-1 (nmol/L)	18.3 (1.3)	17.9 (1.2)	−0.4 [−2.6;1.8]	17.9 (1.4)	19.6 (1.3)	1.6 [−0.7;3.9]	17.1 (1.2)	16.5 (1.1)	−0.6 [−2.7;1.4]	0.13	0.82

Abbreviations: *PBR* perfused boundary region, *MVHS* microvascular health score, *BMI* body mass index, *HDL* high density lipoprotein, *LDL* low density lipoprotein, *Hba1c* hemoglobin A1c, *eGFR CKD-EPI* estimated glomerular filtration rate according to chronic kidney disease epidemiology collaboration, *hsCRP* high sensitivity c-reactive protein, *IGF-1* Insulin growth factor 1, *ns* non-significant; Estimated marginal means with standard error.

^a^Linear mixed models adjusted for age, sex, microvascular history at baseline, macrovascular history at baseline and hypertension at baseline with Bonferroni post-hoc, baseline compared to 3 months, ^b^interaction term of the groups with measurement over time (Baseline-3 months), *p* < 0.05 is considered significant **(bold)**.

### FMD effect on microvascular health

During the monthly 5-day diet cycle, daily glucose measurements (self-measurement) resulted in 2 reported occurrences of hypoglycemia (<4.0 mmol/L) upon continued use of SU-derivates or long acting insulin, of which one patient discontinued due this occurrence of hypoglycemia. Overall, patients experienced less of the time the feeling of hyper- or hypoglycemia after the diet cycles (DTSQ questionnaire, supplementary Table 2). On day 5 of the first diet cycle, in only 3 of 15 patients, ketone bodies could be detected in urine and in 5 patients (of 13) capillary ketone levels of ≥0.5 mmol/L (ketosis) were detected.

**Fig. 2:**
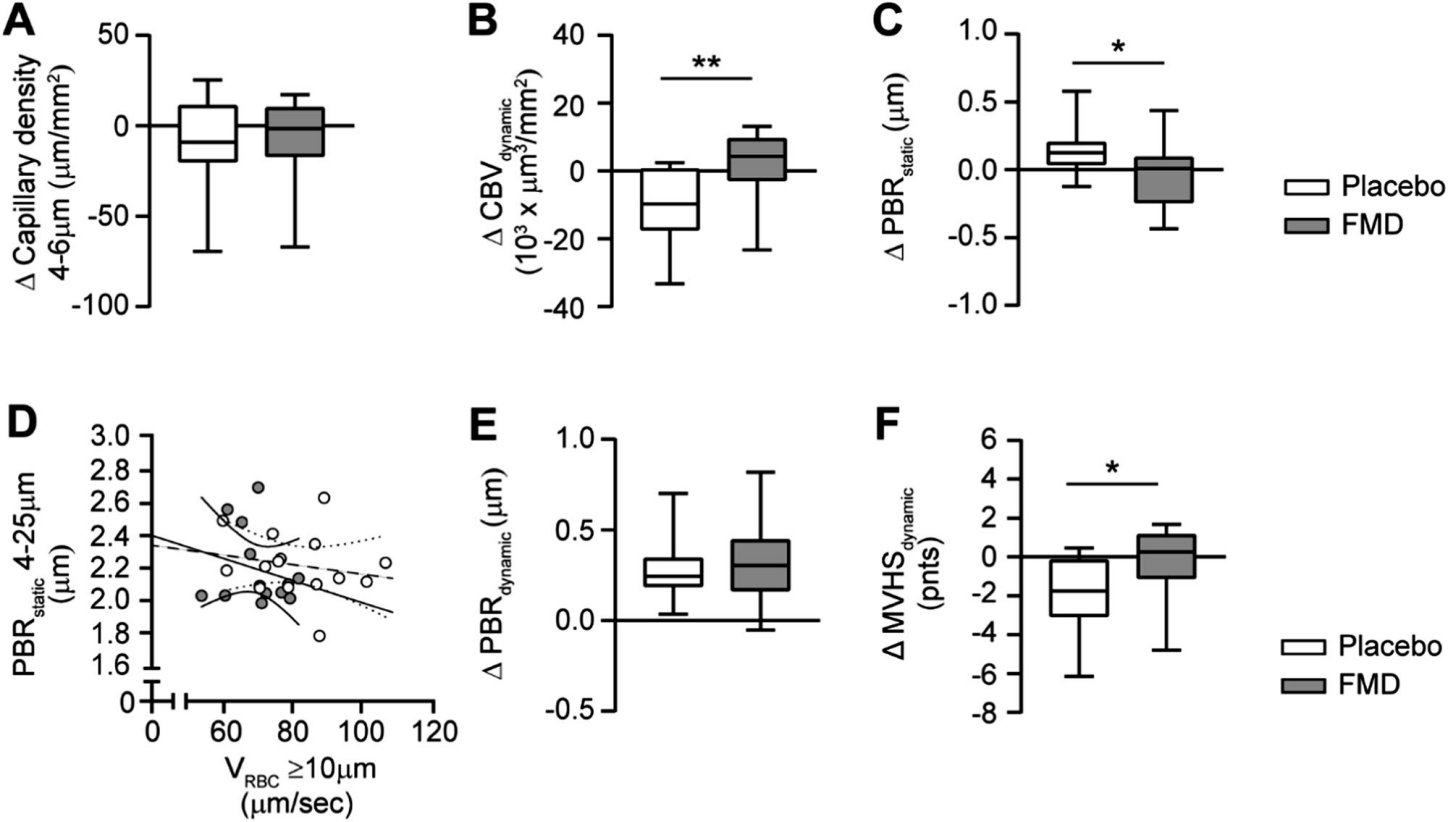
Changes in microvascular parameters upon FMD treatment. Comparison of changes within 3 months intervention between the diet and placebo group of **A** Capillary density, **B** Dynamic capillary blood volume (CBV_dynamic_) and **C** Static perfusion boundary region (PBR_static_), **D** Scatter dot plots and simple linear regression (slope) with 95% confidence intervals of PBR_static_ plotted against red blood cell velocity (V_RBC_) in feed vessels. Comparison of changes after 3 months intervention between diet (continuous line) and placebo (dashed line) of **E** dynamic perfusion boundary region (PBR_dynamic_) and **F** dynamic Microvascular Health Score (MVHS_dynamic_). Box plot whiskers indicate 5th and 95th percentiles. Delta changes were compared between the diet and placebo group with an unpaired t-test. *P < 0.05, **P < 0.01.

Intention to treat analysis using linear mixed model analysis adjusted for age, sex, microvascular or macrovascular complications at baseline and hypertension revealed that after 3 months of recurrent FMD, 3 weeks after last cycle, only PBR_dynamic_ significantly increased (estimated mean difference 0.32 µm (95% CI 0.18–0.50; Table 2). Treatment effects between FMD and placebo groups revealed significant differences within 3 months for CBV_dynamic_, PBR_static_, and MVHS_dynamic_ (Table 2). This was also shown with the per protocol analysis showing the delta differences in microvascular parameters between FMD and placebo in Fig. 2.

While BP, fasting glucose, hsCRP and cholesterol levels were not affected after the diet cycles, mean BMI and serum HbA1c levels were significantly lower compared to baseline with an estimated difference of −1.0 kg/m^2^ (95% CI −1.5−0.5) and −5.1 nmol/L (95% CI −9.6−0.6), respectively. Markers related to glycocalyx degradation (HPSE-1) and endothelial and glycocalyx shedding markers (ANG2 and sTM) were not affected (Table 3).

**Table 3:** Differences between baseline and after 3 months in the interventions groups and treatment effects.

	Placebo			Diet			Supplement				
	Baseline	3 months	Difference [95% CI]^a^	Baseline	3 months	Difference [95% CI]^a^	Baseline	3 months	Difference [95% CI]^a^	Treatment effect diet^b^	Treatment effect supplement^b^
	(*n* = 16)	(*n* = 15)		(*n* = 18)	(*n* = 13)		(*n* = 19)	(*n* = 17)			
**Glycocalyx plasma markers**											
HPSE-1 activity. plasma (U/mL)	1.04 (0.16)	1.54 (0.19)	0.50 [−0.01;1.00]	1.33 (0.17)	1.46 (0.21)	0.12 [−0.41;0.65]	1.27 (0.14)	1.54 (0.17)	0.28 [−0.18;0.74]	0.21	0.43
HYAL-1 activity (U/mL)	0.56 (0.02)	0.54 (0.01)	−0.01 [−0.06;0.03]	N/A	N/A	N/A	0.48 (0.02)	0.52 (0.01)	0.04 [−0.01;0.07]	N/A	**0.05**
HYAL-4 activity (U/mL)	25.10 (2.92)	21.47 (2.6)	−3.63 [−8.78;1.52]	N/A	N/A	N/A	17.82 (2.51)	17.55 (2.17)	−0.27 [−4.87;4.33]	N/A	0.23
HYAL-4 protein (ng/mL)	3.45 (0.41)	3.51 (0.39)	0.07 [−0.27;0.41]	N/A	N/A	N/A	2.79 (0.35)	2.61 (0.33)	−0.17 [−0.48;0.13]	N/A	0.19
ANG-2 protein (ng/mL)	2.47 (0.24)	2.40 (0.21)	−0.06 [−0.39;0.27]	2.77 (0.24)	2.59 (0.23)	−0.18 [−0.51;0.16]	2.82 (0.21)	3.04 (0.18)	0.22 [−0.08;0.52]	0.55	0.12
sTM protein (ng/mL)	5.13 (0.60)	5.80 (0.69)	0.67 [−0.16;1.50]	5.37 (0.62)	5.25 (0.72)	−0.12 [−1.00;0.76]	6.57 (0.52)	6.79 (0.60)	0.22 [−0.53;0.98]	0.11	0.33
**Urinary markers**											
ACR. urine (mg/mmol)	3.1 (2.1)	2.6 (0.7)	−0.5 [−5.5;4.4]	2.7 (2.0)	1 (0.8)	−1.7 [−6.4;3.0]	5.0 (1.9)	2.4 (0.6)	−2.6 [−7.2;1.9]	0.68	0.44
HPSE-1 activity. urine (U/mL)	0.96 (0.19)	0.81 (0.14)	−0.15 [−0.53;0.24]	1.19 (0.21)	0.89 (0.16)	−0.30 [−0.71;0.12]	0.87 (0.17)	0.83 (0.13)	0.04 [−0.40;0.2]	0.52	0.62
MCP-1 activity. urine (ng/mmol)	22.3 (9.7)	39.3 (8.6)	17.1 [−10.2;44.3]	36.6 (9.3)	28.5 (9.4)	−8.10 [−35.7;19.5]	39.3 (8.7)	25.9 (7.8)	−13.4 [−38.3;11.5]	0.11	**0.05**

*HPSE-1* heparanase-1*, HYAL-1* hyaluronidase-1*, HYAL-4* hyaluronidase-4*, ANG-2* Angiopotein-2*, sTM* soluble thrombomodulin*, ACR* albumin creatinine ratio, *MCP*-1 monocyte chemoattractant protein 1, *ns* non-significant; Estimated marginal means with standard error.

^a^Linear mixed models adjusted for age, sex, microvascular complications at baseline, macrovascular complications at baseline and hypertension at baseline with Bonferroni post-hoc, baseline compared to 3 months, ^b^interaction term of the groups with measurement over time (Baseline-3 months), *p* < 0.05 is considered significant (**bold**).

The follow-up measurements at month 6 revealed that BMI was still lower compared to baseline, with a difference of −0.6 kg/m^2^ (95% CI −1.2–0.00). However the effect on Hba1c was lost after 6 months compared to baseline with a difference of −1.8 mmol/mol (95% CI −6.2–2.8) (supplementary Table 1). In contrast, PBR_dynamic_ was increased with a positive linear regression slope (supplementary Fig. 3B) arguing for a continued adverse effect on endothelial function.

### Endocalyx effects on microvascular health

**Fig. 3:**
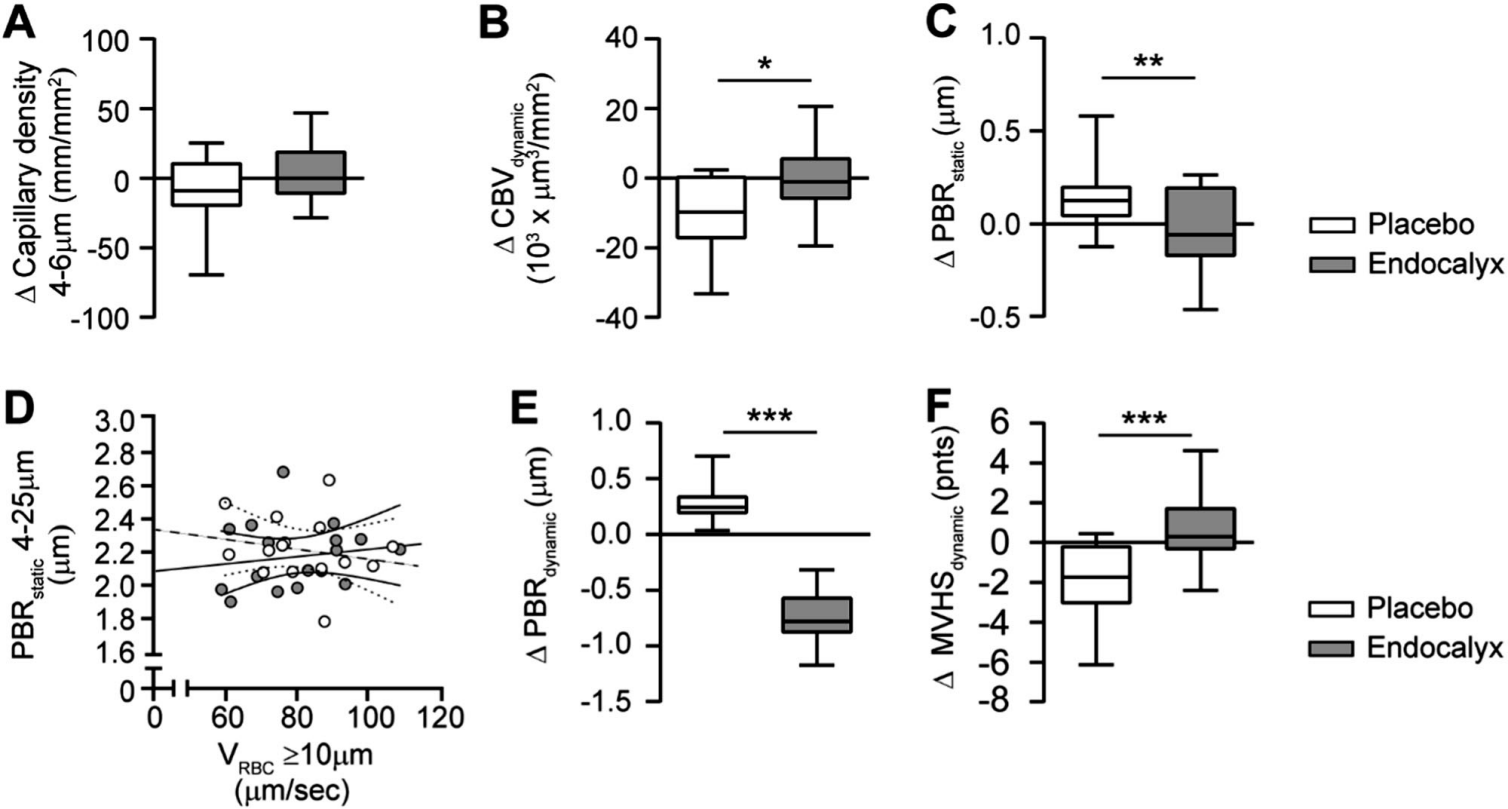
Changes in microvascular parameters upon supplement treatment. Comparison of changes within 3 months intervention between the supplement and placebo of **A** Capillary density, **B** Dynamic capillary blood volume (CBV_dynamic_) and **C** Static perfusion boundary region (PBR_static_), **D** Scatter dot plots and simple linear regression (slope) with 95% confidence intervals of PBR_static_ plotted against red blood cell velocity (V_RBC_) in feed vessels. Comparison of changes after 3 months intervention between supplement (continuous line) and placebo (dashed line) of **E** dynamic perfusion boundary region (PBR_dynamic_) and **F** dynamic Microvascular Health Score (MVHS_dynamic_). Box plot whiskers indicate 5th and 95th percentiles. Delta changes were compared between the supplement and placebo group with an unpaired t-test. *P < 0.05, **P < 0.01, ***P < 0.0001.

After 3 months of daily supplementation with Endocalyx, a significant improvement in the PBR_dynamic_ (estimated difference of −0.73 µm; 95% CI −0.85 – −0.61) and MVHS_dynamic_ of 0.7 points (95% CI −0.3–1.7) was seen, while capillary density, CBV and PBR_static_ did not change significantly (Table 2). Treatment effects between Endocalyx and placebo groups for the intention to treat and per protocol analysis showed to be significant for CBV_dynamic_, PBR_static_, PBR_dynamic_ and MVHS_dynamic_ confirming an overall improvement in vascular health after 3 months of glycocalyx mimetics supplementation (Fig. 3 and Table 2). The follow-up measurements at month 6 revealed a continuation in improved microvascular health (supplementary Fig. 3C and supplementary Table 1).

Markers related to glycocalyx degradation (HPSE-1, HYAL-1 and HYAL-4) and endothelial and glycocalyx shedding markers (ANG2 and sTM), however, were not affected (Table 3). Overall, ACR did not significantly change (estimated difference of −2.6 mg/mmol, 95% CI −7.2–1.9), although in two patients with the highest baseline ACR (28.8 and 37.7 mg/mmol, respectively) albuminuria decreased to normo-albuminuric levels after 3 months of Endocalyx. Within this group, urinary MCP-1 activity did not significantly change within 3 months (estimated difference of −13.4 ng/mmol with 95% CI −38.3–11.5), although compared to placebo urinary MCP-1 activity was significantly different (*p* = 0.05) (Table 3). No significant changes in other clinical or laboratory parameters were observed (Table 2).

## Discussion

We evaluated the effect of 2 short-term dietary interventions on microvascular endothelial health in SA-T2DM. Overall, we found that capillary density in this patient population was already lower than expected compared to earlier measurements within the NEO study [26], and seems in line with the observed existing cardiovascular problems these patients are facing [3]. We also observed in the placebo that during the first 3 months microvascular parameters worsened.

After 3 recurrent FMD cycles, PBR_dynamic_ alone increased, which continued to worsen at 3 months after the last diet cycle. We observed a significant reduction in plasma HbA1c levels and BMI in the first 3 months and the effect on BMI was still present after discontinuation of FMD.

Supplementation with Endocalyx for 3 months, however, showed an improvement in the microvascular parameters CBV_dynamic_, PBR_static_, PBR_dynamic_ and MVHS_dynamic_ which was also reflected in the linear regression slopes (PBR_static_ vs. V_RBC_). Even 3 months after the intervention this effect was still present, indicating a possible legacy effect. Interestingly, we found improvement of albuminuria in 2 patients in this group which was accompanied by an improved PBR. The observed significantly reduced urinary MCP-1 activity after 3 months, however, was lost at follow-up. No effect on the other clinical or laboratory markers were observed upon Endocalyx supplementation.

Although in early diabetic nephropathy inhibition of HPSE-1 activity has been shown to protect the endothelial glycocalyx and to prevent development of proteinuria [15, 36], we could not demonstrate this effect in the present study. Following this study, we recently observed ethnic differences in urinary HPSE-1 and MCP-1 activity in individuals with T2DM from the HELIUS study [37]. Interestingly, the South-Asian Surinamese participants showed the lowest urinary HPSE-1 activity. Urinary HPSE-1 activity in this study was only statistically significant in participants of Dutch origin in relation to ACR.

Previous diet intervention studies in patients with type 2 diabetes revealed reduced BMI, blood pressure, fasting glucose, total and low density cholesterol, CRP and IGF-1 levels in a cohort of 100 participants, particularly in those at risk for disease [38–40]. We recently showed in an experimental diabetic study, that a repeated FMD was able to partially preserve glomerular endothelial glycocalyx coverage, however, perturbing glomerular metabolic responses resulting in increased oxidative stress and reduced catabolic breakdown products [23]. While capillary loop morphology and endothelial glycocalyx heparan sulfate contents was preserved, hyaluronan surface expression was reduced which coincided with reduced UDP-glucuronic acid, a rate limiting building block in its biosynthesis. Despite the positive effects in the first FMD trial in patients with T2DM [22], showing beneficial effects on albuminuria levels in patients with micro-albuminuria next to beneficial effects on HbA1c levels and BMI, the lack of improvement in inflammatory or glycocalyx degrading markers in our study could be due to the already established cardiovascular problems, such as retinopathy, neuropathy and other cardiovascular disease markers. These cardiovascular comorbidities, however, do not seem to correspond with the observed albuminuria and these observations argue for alternative disease biomarkers in this specific patient population.

The main risk of severe calorie restriction in patients with diabetes is hypoglycemia and several studies on fasting regimes in T2DM patients emphasize the importance of adjusting the dosage of glucose lowering medications during fasting days [38, 41]. In our study, the dosages of the sulfonylurea derivatives and insulins were changed or stopped during the 5-day fasting cycle to minimize the chance of hypoglycemia. This resulted still in 2 reported occurrences of hypoglycemia during the FMD cycles while patients did experience less of the time the feeling of hyper- or hypoglycemia after the diet cycles. It appears that, when medication dosage is adapted appropriately and patients are frequently monitored, a low caloric diet can be used safely in patients using sulfonylurea derivatives or insulin.

In one patient, the estimated glomerular filtration rate, which was not routinely measured in this study, turned out to be seriously deteriorated after the first diet cycle. This decline of kidney function appeared to be due to dehydration, as the kidney function fully recovered after intravenous fluid therapy. It has been known for a long time that severe calorie restriction is accompanied by loss of sodium and body water via a largely unexplained mechanism [42, 43]. Therefore, people who fast or use a FMD should always be encouraged to drink sufficiently. In our experimental study, we also found that weight loss during the FMD was mainly due to loss of water and lean mass [23]. Currently, a clinical trial with this diet in patients with diabetes is investigating the effect on body composition by collecting MRI data after a diet cycle [44]. For now, it might be advised that FMD should be used with caution in patients with decreased kidney function or CKD and in patients using diuretics, where kidney function should be monitored regularly.

This study has several limitations. Firstly, the low sample size and drop-out rate of the study. We experienced a low response rate in patients that were contacted for the clinical study. Conducting lifestyle intervention studies in the South-Asian population has been proven to be extremely difficult due to low response rates, high drop-out rates and lack of effect on lifestyle [45, 46]. We experienced a drop-out rate of 30% in the diet group, comparable to other FMD studies [40]. In addition, due to the COVID-19 epidemic, inclusions of the study had to be discontinued. The low sample size may have reduced the power to show significant effects in the intervention groups although still clear primary endpoints were observed. Secondly, in only a few patients after the FMD cycle, capillary or urinary ketone levels were elevated reflecting ketosis. During fasting, when glycogen stores are depleted and glucose is less available, fatty acids are released from adipose tissue to serve as an alternative fuel, and excessive oxidation of fatty acids is accompanied by ketogenesis. These patients may not have been compliant with the dietary regime. Another explanation, however, could be that the switch from carbohydrate to lipid oxidation in response to fasting is impaired in South-Asians (as compared to European) with T2DM, reflecting metabolic inflexibility in South-Asian individuals [47]. As ketones are probably involved in the health effects of fasting [48], this may indicate that the benefits of fasting or fasting mimicking diets are less effective in individuals from South-Asian descent than in individuals of European descent.

A strength of the study is its execution in general practitioner offices with South-Asian Surinamese patients, a patient group that can be difficult to recruit for clinical trials as mentioned above. An intensive 1 and 2 year targeted lifestyle intervention in general practice revealed no significant weight loss or improvement in metabolic profiles in South-Asian Surinamese participants in The Hague [45, 46]. Although we also experienced low response rates and drop-outs, we did see beneficial effects on BMI and HbA1c levels, already at short term which suggest that South-Asian individuals may find it easier to adhere to repeated short term fasting cycles than continuous caloric restriction or continuous lifestyle interventions.

The development of diabetic vascular complications is a multifactorial pathway, therefore, therapies that target different factors in the pathogenesis are strongly recommended for treatment of diabetes [49]. Preserving endothelial function is seen as a valuable pharmacological target for protecting the microvasculature and reducing the incidence of complications in patients with diabetes [50, 51]. In the present study, we showed that supplementation with Endocalyx^TM^ is a potential candidate able to improve microvascular health in SA-T2DM. We failed to demonstrate direct beneficial microvascular effects of FMD but saw temporarily improvement of metabolic risk factors in SA-T2DM. We confirm that FMD can probably be safely used in patients with diabetes glucose lowering drugs if the dose of these medications are adapted appropriately, but it needs to be used with caution in patients with CKD until further studies has been done.

### Supplementary information

Supplement

## Data Availability

The data underlying this article will be shared on reasonable request to the corresponding author.
