# Supplementary material for: Role of dietary interventions on microvascular health in South-Asian Surinamese people with type 2 diabetes in the Netherlands: A randomized controlled trial
Source: Nutr Diabetes. 2024 Apr 10;14:17. doi: 10.1038/s41387-024-00275-5 (PMC11006941; doi:10.1038/s41387-024-00275-5)
Supplement: Supplementary file 1 — Supplement [file 41387_2024_275_MOESM1_ESM.docx]

**Supplemental data**

**Role of dietary interventions on microvascular health in South-Asian Surinamese people with type 2 diabetes in the Netherlands; a randomized controlled trial**

*Anouk IM van der Velden^1,2^, Daphne HT IJpelaar^3^, Prataap K Chandie Shaw^4^, Hanno Pijl^2,5^, Hans Vink^6,7^, Johan van der Vlag^8*^* *, Ton J Rabelink^1,2*^* , *Bernard M van den Berg,^1,2*^*

^1^ Department of Internal Medicine (Nephrology), Leiden University Medical Center, Leiden, The Netherlands.
^2^ Einthoven Laboratory of Vascular and Regenerative Medicine, LUMC, Leiden, The Netherlands
^3^ Department of Internal Medicine and Nephrology, Green Heart Hospital, Gouda, The Netherlands
^4^ Department of Internal Medicine and Nephrology, Haaglanden Medical Center, The Hague, The Netherlands
^5^ Department of Internal Medicine (Endocrinology), LUMC, Leiden, The Netherlands
^6^ Department of Physiology, Cardiovascular Research Institute Maastricht, Maastricht, The Netherlands
^7^ MicroVascular Health Solutions LLC, Alpine, Utah, USA
^8^ Department of Nephrology, Radboud University Medical Center, Nijmegen, The Netherlands

*Share last authorship

ORCID: Anouk IM van der Velden (0000-0001-7199-6026), Daphne HT IJpelaar (0000-0002-6721-4121), Prataap K Chandie Shaw (0000-0002-7678-8637), Hanno Pijl (0000-0002-3076-1551), Hans Vink (0000-0001-7517-6535), Johan van der Vlag (0000-0001-7843-5918), Ton J Rabelink (0000-0001-6780-5186), Bernard M van den Berg (0000-0002-5726-5777)

**Supplemental results**

**Effect of the fasting mimicking diet, continued**In all patients except one, HbA1c levels decreased after the FMD cycles compared to baseline levels. In 2 patients, dosages of anti-diabetic medication were lowered in the 3 month intervention period due to improved fasting glucose levels. In 2 patients (one patient of which HbA1c levels did not decrease), the dosage of anti-diabetic medication was increased by the GP (data not shown). The change in BMI and HbA1c between the FMD and placebo group was significantly different as shown by the treatment effects (table 2).
IGF-1 levels did not change after 3 FMD cycles (table 2). Most likely because the study visit took place 3 weeks after the last FMD cycle at the end of 3 month study, resulting in IGF-1 levels already returned to baseline [1]. Furthermore, plasma levels of angiopoietin-2 (ANG2) and soluble thrombomodulin (sTM), and heparanase-1 (HPSE-1) activity, all markers of endothelial activation and glycocalyx degradation did not change after 3 months of FMD cycles (table 2). Nor did the urinary markers such as the ACR, urinary HPSE-1 and MCP-1 activity levels decrease significantly after 3 FMD cycles (table 2).

**FMD treatment satisfaction**The Diabetes Treatment Satisfaction Questionnaire (DTSQ) [2] which was developed to assess the diabetes treatment satisfaction and consists of eight items, each rated on a seven-point scale. The items include satisfaction with current treatment, treatment convenience, flexibility of treatment, understanding of diabetes, continuity of treatment, recommending treatment to others and two additional items about perceived frequency of hyper- and hypoglycemia. The DTSQ static (DTSQs) was conducted at baseline and the items range from 6 (very satisfied) to 0 (very dissatisfied) with the sum of scores ranging from 36 to 0. The perceived frequency of hyper- and hypoglycemia were also scored from 0 (none of the time) to 6 (most of the time). The DTSQ change (DTSQc) was conducted after 3 months of the intervention. DTSQc uses the same eight items as the DTSQs, but assess changes in the treatment satisfaction with their current treatment (intervention) compared to their previous treatment. The items are scored from +3 (much more satisfied now) to -3 (much less satisfied now), with 0 representing no change. The sum of the score thus ranges from 18 to -18. The perceived frequency of hyper- and hypoglycemia are also scored from +3 (much more of the time now) to -3 (much less of the time now).

In both the FMD and placebo group, satisfaction with their current diabetes treatment at the general practitioners office at baseline was high, with a mean DTSQ satisfaction score of 30.3 (± 5.0) in the FMD group and 30.2 (± 4.4) in the placebo group (supplemental table 1). Perceived frequency of hyperglycemia was comparable between both groups as baseline, with a mean score of 2.9 (± 1.7) in the FMD group and 2.9 (± 2.2) in the placebo group. Perceived frequency of hypoglycemia was also comparable between both groups, with a mean score of 1.8 (± 2.0) in the FMD group and 1.6 (± 2.0) in the placebo group.
After the 3 FMD cycles, mean treatment satisfaction score with the DTSQc was 11.3 (±4.5), indicating more satisfaction with the intervention compared to the baseline anti-diabetic medication. The placebo group received placebo capsules for 3 months and were also more satisfied with the intervention, with a mean DTSQc treatment satisfaction score of 10.1 (± 7.5). However, perceived hyper- or hypoglycemia did not change in the placebo group with mean scores of 0.1 on both items. After the 3 FMD cycles, mean score of perceived hyperglycemia was -1.0 (± 1.5), indicating that these patients had less of the time the feeling of hyperglycemia after the FMD. These patients also had less of the time perceived hypoglycemia, with a mean score of -0.5 (± 2.0) on this item after 3 FMD cycles.

**Adverse events FMD**The Common Terminology Criteria for Adverse Events (CTCAE) was used to report adverse event during or after the FMD cycles.The most reported adverse events during the FMD cycles were grade 1 (mild) or grade 2 (moderate) symptoms of headache, fatigue, diarrhea and hypoglycemia. Two patients self-reported the occurrence of hypoglycemia during an FMD cycle . A total of 4 patients dropped out during or after the first cycle due to adverse events. In one patient, a SAE occurred. Kidney function acutely deteriorated at day 5 of the first FMD cycle as compared to previous measurements, which was noticed during a routine check at the hospital. The creatinine level at baseline was 125 µmol/L for this patient, which increased to 169 µmol/L at day 5 of the FMD and increased even further one week later to 202 µmol/L. The patient was hospitalized and rehydrated, after which the creatinine levels normalized to baseline values. This patient was withdrawn from the study by the investigator. No negative effects were seen on kidney function 3 weeks after the third FMD cycle (table 2).

**References**

1. Caputo, M., et al., *Regulation of GH and GH Signaling by Nutrients.* Cells, 2021. **10**(6).

2. Bradley, C., *Diabetes treatment satisfaction questionnaire. Change version for use alongside status version provides appropriate solution where ceiling effects occur.* Diabetes Care, 1999. **22**(3): p. 530-2.

3. van der Velden, A.I.M., et al., *Microvascular differences in individuals with obesity at risk of developing cardiovascular disease.* Obesity (Silver Spring), 2021. **29**(9): p. 1439-1444.


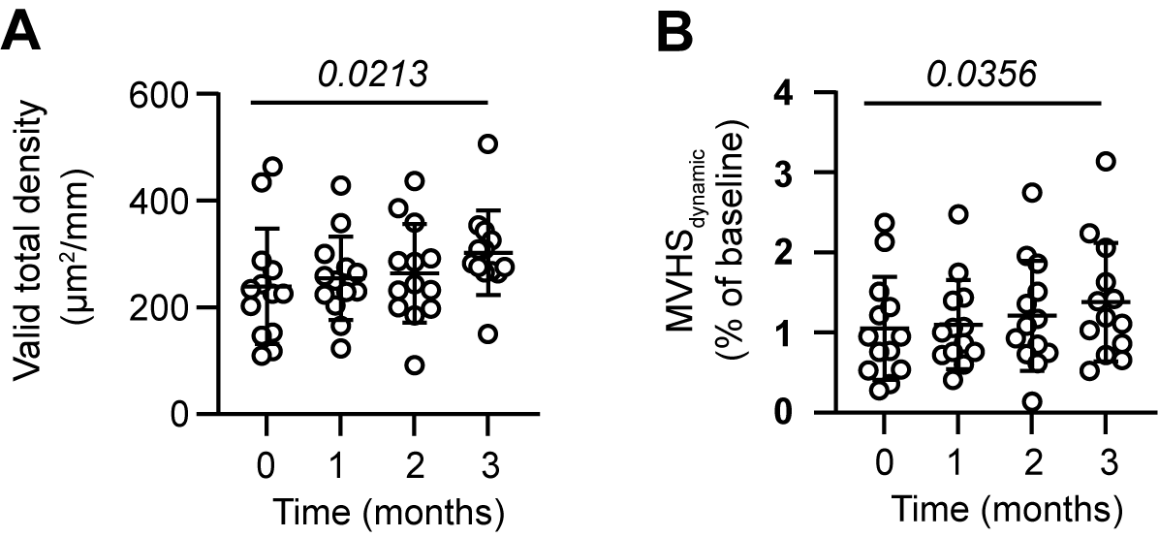


**Supplemental figure 1. Pilot study to determine minimal sample size necessary.** Healthy volunteers were given the food supplement Endocalyx^TM^ for 3 consecutive months (n = 13 final measurements) revealing **(A)** valid total microvascular density, and **(B)** overall microvascular health score (MVHS_dynamic_). Significance over the three month supplementation is given and was performed using one-way ANOVA (+ Geiser-Greenhouse correction) with mixed effects-model and Dunnet multiple comparison test. After 3 months, a 31% improvement in MVHS_dynamic_ was demonstrated, expressed in a significant increase of from 1.05 at baseline, to 1.38 at 3 months (improvement of 0.33). To achieve per group a 80% power to detect a difference of 0.35 between the null hypothesis that group improvement means are 0.0 and the alternative hypothesis that the improvement mean of group 2 or 3 is 0.35 with group standard deviations of 0.41 and a significance level (alpha) of 0.05 (two-sided two-sample t-test) the estimated group sample size is 23.


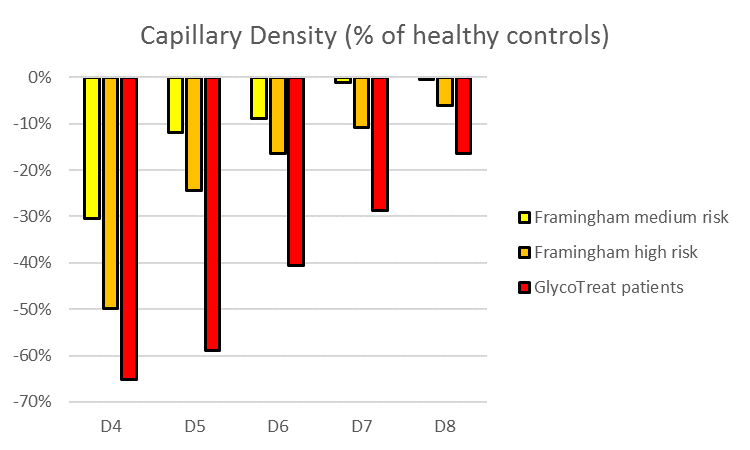


**Supplemental figure 2. Comparison capillary density loss in South Asian patients with T2DM at baseline.** Percentage difference in the number of perfused capillaries (capillary density) per capillary diameter group of total South Asian patients with T2DM in current study at baseline (n = 53) and from intermediate- and high-risk Framingham groups (n = 253 or 168, respectively) from the published NEO study[3] were compared to the low-risk Framingham group (as reference, n = 392).


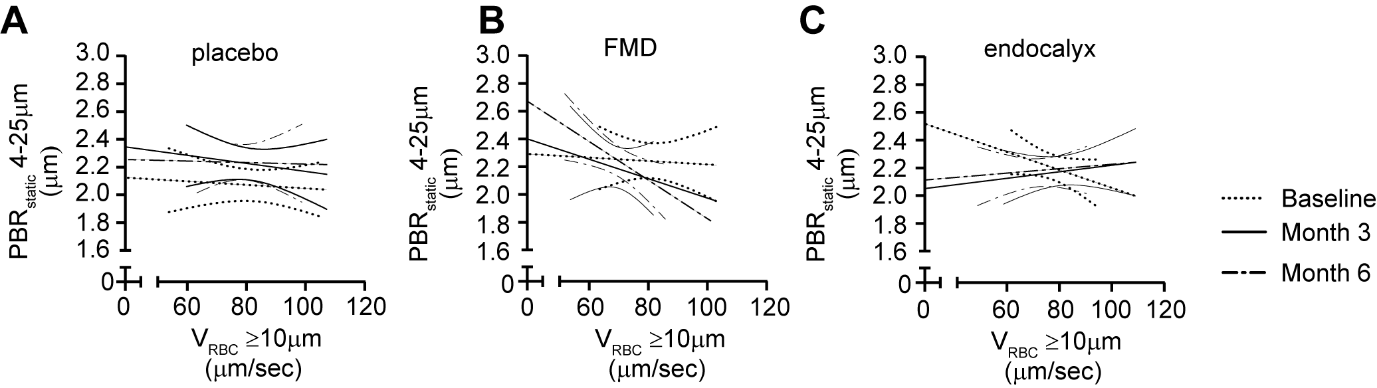


**Supplemental figure 3. Changes in linear regression slopes over time.** Comparison of linear regression slopes with 95% confidence intervals of PBR_static_ plotted against red blood cell velocity (V_RBC_) in feed vessels at each consecutive timepoint of **(A)** placebo, **(B)** diet (FMD) and **(C)** supplement (endocalyx) group. Comparison of changes between baseline, after intervention at 3 months and at month 6 (3 months after last intervention).

**Supplemental table 1. Differences between baseline and after 6 months and intervention groups in treatment effects.**

|  | Placebo | | | Diet | | | Supplement | | |  |  | |
| --- | --- | --- | --- | --- | --- | --- | --- | --- | --- | --- | --- | --- |
|  | Baseline | 6 months | Difference [95% CI]^1^ | Baseline | 6 months | Difference [95% CI]^1^ | Baseline | 6 months | Difference [95% CI]^1^ | Treatment effect  diet^2^ | Treatment effect supplement^2^ |  |
|  | (n=16) | (n=15) |  | (n=18) | (n=13) |  | (n=19) | (n=17) |  |  |  |  |
| **Microvascular parameters** |  |  |  |  |  |  |  |  |  |  |  |  |
| Capillary density (4-6) (µm/mm²) | 43 (6) | 45 (6) | 1 [-16;18] | 32 (6) | 35 (6) | 4 [-14;21] | 32 (5) | 36 (5) | 4 [-12;19] | 0.81 | 0.80 |  |
| Capillary blood volume static (pL/mm^2^) /10^3^ μm^3^ | 12.7 (1.7) | 11.7 (1.5) | -1.0  [-5.7;3.7] | 10.1 (1.7) | 10.4 (1.6) | 0.3 [-4.5;5.0] | 10.0 (1.5) | 11.0 (1.3) | 1.0 [-3.3;5.2] | 0.64 | 0.45 |  |
| Capillary blood volume dynamic (pL/mm^2^) /10^3^ μm^3^ | 21.6 (2.5) | 13.1 (1.9) | **-8.5  [-15.2;-1.8]** | 11.8 (2.5) | 17.3 (2.1) | 5.5  [-1.2;12.2] | 14.7 (2.3) | 14.2 (1.7) | -0.5  [-6.6;5.5] | **0.001** | **0.03** |  |
| PBR static (µm) | 2.08 (0.05) | 2.24 (0.05) | **0.16 [0.01;0.31]** | 2.26 (0.05) | 2.23 (0.06) | -0.03  [-0.18;0.13] | 2.21 (0.04) | 2.17 (0.05) | -0.05  [-0.19;0.09] | **0.04** | **0.02** |  |
| PBR dynamic (µm) | 2.19 (0.05) | 2.27 (0.05) | 0.08  [-0.07;0.24] | 2.35 (0.05) | 3.21 (0.05) | **0.86 [0.70;1.00]** | 2.74 (0.04) | 2.09 (0.04) | -**0.65  [-0.78;-0.51]** | **<0.01** | **<0.01** |  |
| MVHS dynamic (µm) | 3.9 (0.5) | 2.2 (0.3) | **-1.7  [-2.8;-0.5]** | 2.1 (0.5) | 2.1 (0.3) | 0.1 [-1.1;1.2] | 2.2 (0.4) | 2.7 (0.3) | 0.6 [-0.5;1.6] | **0.01** | **0.001** |  |
| **Clinical parameters** |  |  |  |  |  |  |  |  |  |  |  |  |
| Systolic blood pressure (mmHg) | 132 (4) | 135 (3) | 3 [-7;14] | 141 (4) | 131 (3) | -10 [-21;0] | 133 (3) | 130 (3) | -3 [-13;7] | **0.04** | 0.30 |  |
| Diastolic blood pressure (mmHg) | 82 (2) | 85 (2) | 3 [-3;9] | 81 (2) | 78 (2) | -3 [-9;3] | 80 (2) | 80 (2) | -1 [-6;5] | 0.06 | 0.27 |  |
| BMI (kg/m^2^) | 26.4 (1.3) | 26.4 (1.3) | 0 [-0.6;0.5] | 28.3 (1.3) | 27.8 (1.3) | **-0.6 [-1.2;0]** | 30.2 (1.1) | 30 (1.1) | -0.2 [-0.7;0.3] | 0.12 | 0.61 |  |
| **Laboratory markers** |  |  |  |  |  |  |  |  |  |  |  |  |
| Fasting glucose (mmol/L) | 7.3 (0.5) | 7 (0.6) | -0.3  [-1.4;0.8] | 7.2 (0.5) | 7.6 (0.6) | 0.4 [-0.8;1.6] | 8.3 (0.4) | 8.1 (0.5) | -0.1  [-1.1;0.9] | 0.30 | 0.79 |  |
| HbA1c (%)  (mmol/mol) | 55.6 (3.3) | 56.9 (3.4) | 1.3 [-3;5.6] | 55.3 (3.4) | 53.6 (3.6) | -1.8  [-6.3;2.8] | 57.9 (2.9) | 59.2 (3) | 1.3 [-2.7;5.2] | 0.23 | 0.98 |  |
| C-peptide (nmol/L) | 1.2 (0.1) | 1.3 (0.2) | 0.1 [-0.1;0.3] | 1.4 (0.1) | 1.5 (0.2) | 0.1 [-0.2;0.3] | 1.5 (0.1) | 1.6 (0.1) | 0.1 [-0.1;0.3] | 0.83 | 0.97 |  |
| eGFR CKD-EPI (ml/min/1.73m^2^) | 86 (5) | 84 (4) | -2 [-8;4] | 83 (5) | 84 (4) | 1 [-5;7] | 80 (4) | 82 (4) | 2 [-4;7] | 0.42 | 0.27 |  |
| Total cholesterol (mmol/L) | 4.03 (0.23) | 4.09 (0.23) | 0.06  [-0.38;0.49] | 4.34 (0.24) | 4.31 (0.25) | -0.03  [-0.48;0.42] | 4.35 (0.2) | 4.28 (0.2) | -0.07  [-0.46;0.32] | 0.73 | 0.59 |  |
| HDL-cholesterol (mmol/L) | 1.27 (0.08) | 1.31 (0.09) | 0.05  [-0.06;0.15] | 1.22 (0.08) | 1.24 (0.09) | 0.02  [-0.09;0.13] | 1.08 (0.07) | 1.06 (0.07) | -0.02  [-0.11;0.08] | 0.71 | 0.28 |  |
| LDL-cholesterol (mmol/L) | 2.34 (0.2) | 2.40 (0.18) | 0.06  [-0.31;0.44] | 2.4 (0.21) | 2.22 (0.20) | -0.18  [-0.57;0.21] | 2.41 (0.18) | 2.32 (0.16) | -0.09  [-0.44;0.26] | 0.27 | 0.46 |  |
| Triglycerides (mmol/L) | 1.11 (0.5) | 1.15 (0.33) | 0.04  [-0.65;0.73] | 1.65 (0.5) | 1.84 (0.35) | 0.19  [-0.5;0.88] | 2.31 (0.45) | 2.17 (0.29) | -0.14  [-0.77;0.49] | 0.69 | 0.64 |  |
| hsCRP (mg/L) | 4.0 (1.1) | 1.8 (1.2) | -2.3  [-4.9;0.3] | 3.6 (1.1) | 3.1 (1.3) | -0.5  [-3.1;2.2] | 6.0 (1.0) | 6.9 (1.0) | 0.9 [-1.4;3.2] | 0.23 | **0.03** |  |
| IGF-1 (nmol/L) | 18.3 (1.3) | 19.6 (1.4) | 1.3 [-1.5;4.2] | 17.9 (1.4) | 18.7 (1.5) | 0.8 [-2.2;3.7] | 17.1 (1.2) | 17.5 (1.2) | 0.4 [-2.2;2.9] | 0.72 | 0.54 |  |

Abbreviations: *PBR* perfused boundary region, *MVHS* microvascular health score, *BMI* body mass index, *HbA1c* hemoglobin A1c, *eGFR CKD-EPI* estimated glomerular filtration rate according to chronic kidney disease epidemiology collaboration, *HDL* high density lipoprotein, *LDL* low density lipoprotein, *hsCRP* high sensitivity c-reactive protein, *IGF-1* Insulin growth factor 1, *ns* non-significant; Estimated marginal means with standard error

^1^Linear mixed models adjusted for age, sex, microvascular history at baseline, macrovascular history at baseline and hypertension at baseline with Bonferroni post-hoc, baseline compared to 6 months, ^2^interaction term of intervention group with measurement over time (Baseline-6 months), p<0.05 is considered significant (**bold**).

**Supplemental table 2. Diabetes Treatment Satisfaction Questionnaire.**

|  | placebo | diet | p-value^1^ |
| --- | --- | --- | --- |
| Baseline – DTSQ static | n=15 | n=17 |  |
| Treatment satisfaction score | 30.2 (4.4) | 30.3 (5.0) | ns |
| Perceived frequency of hyperglycaemia | 2.9 (2.2) | 2.9 (1.7) | ns |
| Perceived frequency of hypoglycaemia | 1.6 (2.0) | 1.8 (2.0) | ns |
| Month 3 – DTSQ change | n=15 | n=13 |  |
| Treatment satisfaction score | 10.1 (7.5) | 11.3 (4.5) | ns |
| Perceived frequency of hyperglycaemia | 0.1 (1.5) | -1.0 (1.5) | ns |
| Perceived frequency of hypoglycaemia | 0.1 (1.8) | -0.5 (2.0) | ns |

Abbreviations: *DTSQ* Diabetes Treatment Satisfaction Questionnaire, *ns* non-significant
^1^Unpaired t-test, P<0.05 is considered significant. 1 missing baseline in both diet and placebo group.

**Supplemental table 3. Differences between baseline and after 6 months and intervention groups in treatment effects.**

|  | Placebo | | | Diet | | | Supplement | | |  |  |
| --- | --- | --- | --- | --- | --- | --- | --- | --- | --- | --- | --- |
|  | Baseline | 6 months | Difference [95% CI]^1^ | Baseline | 6 months | Difference [95% CI]^1^ | Baseline | 6 months | Difference [95% CI]^1^ | Treatment effect diet^2^ | Treatment effect supplement^2^ |
|  | (n=16) | (n=15) |  | (n=18) | (n=13) |  | (n=19) | (n=17) |  |  |  |
| **Glycocalyx plasma markers** |  |  |  |  |  |  |  |  |  |  |  |
| HPSE-1 activity. plasma (U/mL) | 1.04 (0.16) | 0.85 (0.16) | -0.19  [-0.72;0.34] | 1.33 (0.17) | 1.09 (0.18) | -0.24  [-0.78;0.30] | 1.27 (0.14) | 1.23 (0.15) | -0.03  [-0.52;0.45] | 0.87 | 0.58 |
| HYAL-1 activity (U/mL) | 0.56 (0.02) | 0.54 (0.02) | **-0.02  [-0.06;-0.02]** | N/A | N/A | N/A | 0.48 (0.02) | 0.47 (0.02) | **-0.01  [-0.05;-0.02]** | N/A | 0.65 |
| HYAL-4 activity (U/mL) | 25.1 (2.92) | 21.33 (2.64) | -3.77  [-8.39;0.85] | N/A | N/A | N/A | 17.82 (2.51) | 15.86 (2.26) | -1.96  [-6.16;2.24] | N/A | 0.47 |
| HYAL-4 protein (ng/mL) | 3.45 (0.41) | 3.27 (0.39) | -0.18  [-0.46;0.09] | N/A | N/A | N/A | 2.79 (0.35) | 2.67 (0.33) | -0.11  [-0.36;0.14] | N/A | 0.71 |
| ANG-2 protein (ng/mL) | 2.47 (0.24) | 2.57 (0.27) | 0.11  [-0.34;0.56] | 2.77 (0.24) | 2.94 (0.28) | 0.17 [-0.29;0.64] | 2.82 (0.21) | 3.19 (0.24) | 0.36  [-0.05;0.77] | 0.81 | 0.30 |
| sTM protein (ng/mL) | 5.13 (0.60) | 4.29 (0.61) | **-0.83  [-1.49;-0.17]** | 5.37 (0.62) | 4.66 (0.64) | **-0.71  [-1.38;-0.04]** | 6.57 (0.52) | 5.49 (0.53) | **-1.08  [-1.68;-0.48]** | 0.74 | 0.49 |
| **Urinary markers** |  |  |  |  |  |  |  |  |  |  |  |
| ACR. urine (mg/mmol) | 3.1 (2.1) | 2.8 (0.7) | -0.3  [-5.1;4.6] | 2.7 (2.0) | 1 (0.8) | -1.7  [-6.3;2.9] | 5.0 (1.9) | 2.1 (0.6) | -2.9  [-7.4;1.5] | 0.59 | 0.31 |
| HPSE-1 activity. urine (U/mL) | 0.96 (0.19) | 0.55 (0.11) | **-0.41 [-0.83;-0.01]** | 1.19 (0.21) | 0.67 (0.13) | **-0.52  [-095;-0.07**] | 0.87 (0.17) | 0.60 (0.09) | -0.28  [-0.65;0.09] | 0.68 | 0.54 |
| MCP-1 activity. urine (ng/mmol) | 22.3 (9.7) | 20.6 (4.7) | -1.7  [-23.7;20.4] | 36.6 (9.3) | 28.2 (5.3) | -8.5  [-29.6;12.7] | 39.3 (8.7) | 25.9 (4.1) | -13.4  [-33.4;6.7] | 0.59 | 0.34 |

Abbreviations: *HPSE-1* heparanase-1, *HYAL-1* hyaluronidase-*1, HYAL-4* hyaluronidase-4, *ANG-2* Angiopotein-2*, sTM* soluble thrombomodulin*, ACR* albumin creatinine ratio, *MCP*-1 monocyte chemoattractant protein 1, *N/A* not applicable, *ns* non-significant; Estimated marginal means with standard error

^1^Linear mixed models adjusted for age, sex, microvascular history at baseline, macrovascular history at baseline and hypertension at baseline with Bonferroni post-hoc, baseline compared to 6 months, ^2^interaction term of intervention group with measurement over time (Baseline-6 months), p<0.05 is considered significant (**bold**).
